# The genome sequence of the Brown Long-eared bat, *Plecotus auritus *(Linnaeus 1758)

**DOI:** 10.12688/wellcomeopenres.21501.1

**Published:** 2024-05-09

**Authors:** Amanda Millar, Sonja C. Vernes, Emma C Teeling, Meike Mai, Natural History Museum Genome Acquisition Lab, Darwin Tree of Life Barcoding collective, Wellcome Sanger Institute Tree of Life Management, Samples and Laboratory team, Wellcome Sanger Institute Scientific Operations: Sequencing Operations, Wellcome Sanger Institute Tree of Life Core Informatics team, Tree of Life Core Informatics collective, Darwin Tree of Life Consortium

**Affiliations:** 1 Independent researcher, Bat Hospital, Hurstpierpoint, England, UK; 2 School of Biology, University of St Andrews, St Andrews, Scotland, UK; 3 Neurogenetics of Vocal Communication Group, Max Planck Institute for Psycholinguistics, Nijmegen, Gelderland, The Netherlands; 4 School of Biology and Environmental Science, University College Dublin, Dublin, Leinster, Ireland; 5 Wellcome Sanger Institute, Hinxton, England, UK

**Keywords:** Plecotus auritus, Brown Long-eared bat, genome sequence, chromosomal, Chiroptera

## Abstract

We present a genome assembly from a female *Plecotus auritus* (Brown Long-eared bat; Chordata; Mammalia; Chiroptera; Vespertilionidae). The genome sequence is 2163.2 megabases in span. Most of the assembly is scaffolded into 16 chromosomal pseudomolecules, including the X sex chromosome. The mitochondrial genome has also been assembled and is 16.91 kilobases in length.

## Species taxonomy

Eukaryota; Metazoa; Eumetazoa; Bilateria; Deuterostomia; Chordata; Craniata; Vertebrata; Gnathostomata; Teleostomi; Euteleostomi; Sarcopterygii; Dipnotetrapodomorpha; Tetrapoda; Amniota; Mammalia; Theria; Eutheria; Boreoeutheria; Laurasiatheria; Chiroptera; Yangochiroptera; Vespertilionoidea; Vespertilionidae; *Plecotus*; *Plecotus auritus* Linnaeus 1758 (NCBI:txid61862) (subordinal taxonomy updated per Teeling *et al.*, 2005).

## Background

The Brown Long-eared bat, *Plecotus auritus* (Linnaeus 1758), is a small insectivorous bat with slow hovering flight found in and near woodland and hedgerows. Although able to echolocate it seems mainly to use its extremely long ears to listen for prey, and its relatively large eyes, to catch prey such as moths. It also gleans insects and spiders from leaves and bark. It can fold its ears down when at rest ( Swift, 1988).

*Plecotus auritus* is noted as of Least Concern on the IUCN Red List ( IUCN, 2022). It is found across Europe, from the UK in the west, to the Urals in the east. In the UK it regularly roosts in lofts of houses and has a small home range. Because of its slow hovering flight and preference for house lofts and gleaning, it is vulnerable to capture by cats and to being caught in sticky insect traps ( Swift, 1988).

The genome of the Brown Long-eared bat, *Plecotus auritus*, was sequenced as part of the Darwin Tree of Life Project, a collaborative effort to sequence all named eukaryotic species in the Atlantic Archipelago of Britain and Ireland. Here we present a chromosomal-level genome sequence for *Plecotus auritus*, based on a euthanised female specimen from East Sussex, UK.

## Genome sequence report

**Figure 1.**
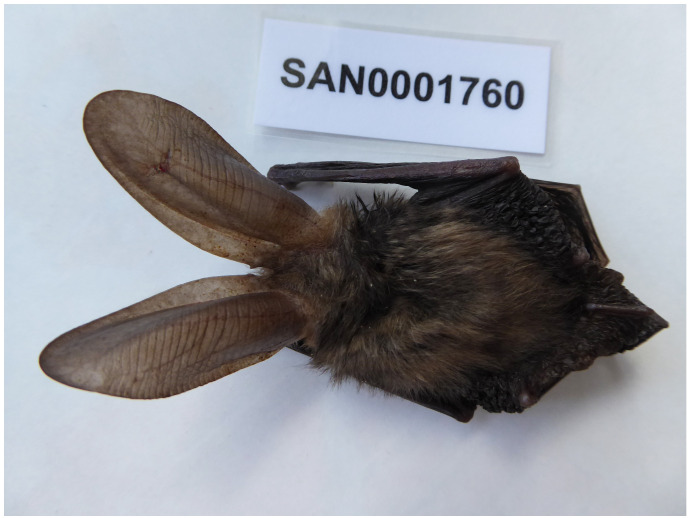
Photograph of the *Plecotus auritus* (mPleAur1) specimen used for genome sequencing.

The genome was sequenced from a female *Plecotus auritus* ( Figure 1) collected from Ringmer, East Sussex, England (50.89, 0.05). A total of 34-fold coverage in Pacific Biosciences single-molecule HiFi long reads was generated. Primary assembly contigs were scaffolded with chromosome conformation Hi-C data. Manual assembly curation corrected 33 missing joins or mis-joins, reducing the scaffold number by 21.69%, and increasing the scaffold N50 by 4.10%.

**Figure 2.**
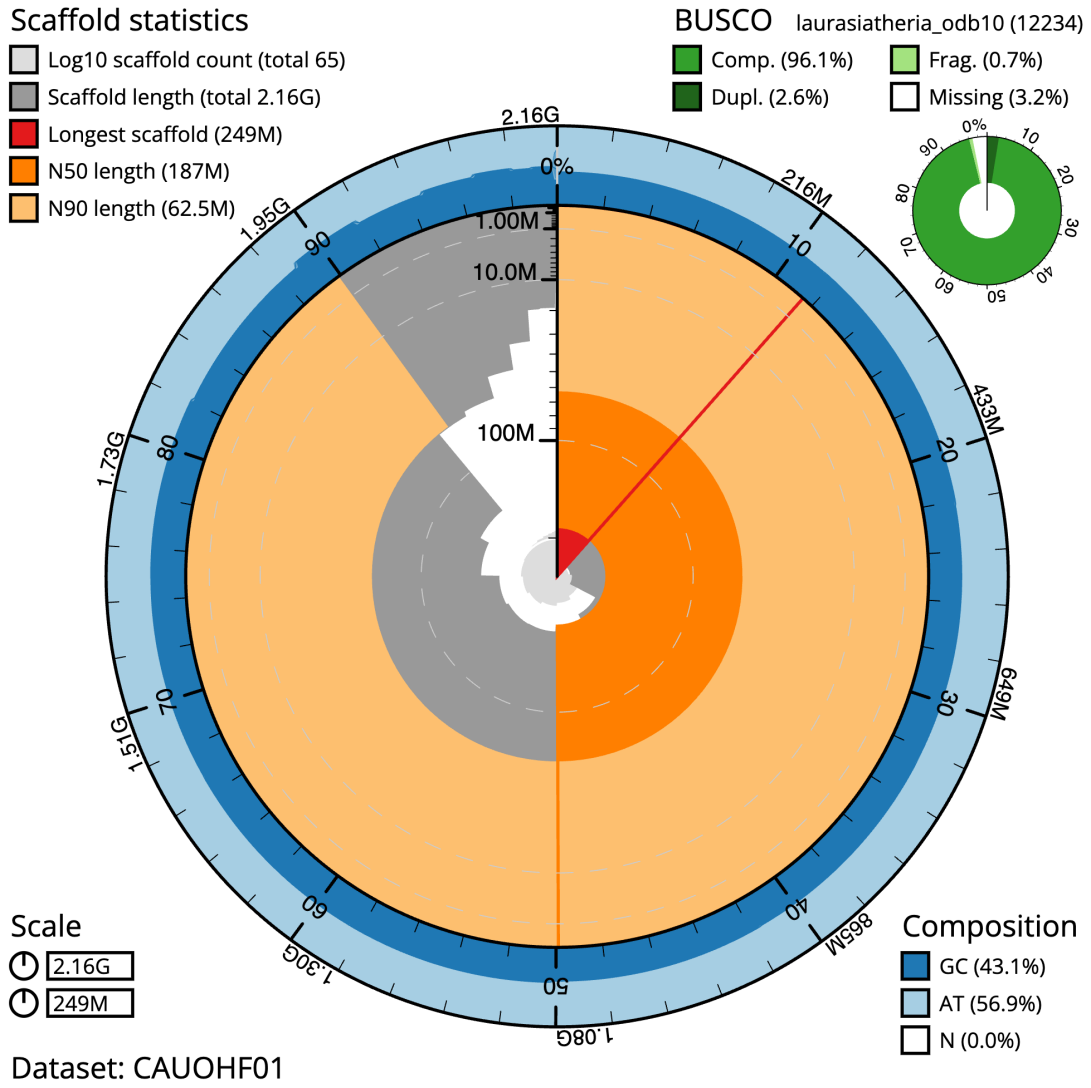
Genome assembly of *Plecotus auritus*, mPleAur1.1: metrics. The BlobToolKit snail plot shows N50 metrics and BUSCO gene completeness. The main plot is divided into 1,000 size-ordered bins around the circumference with each bin representing 0.1% of the 2,163,221,563 bp assembly. The distribution of scaffold lengths is shown in dark grey with the plot radius scaled to the longest scaffold present in the assembly (248,793,850 bp, shown in red). Orange and pale-orange arcs show the N50 and N90 scaffold lengths (186,501,726 and 62,501,937 bp), respectively. The pale grey spiral shows the cumulative scaffold count on a log scale with white scale lines showing successive orders of magnitude. The blue and pale-blue area around the outside of the plot shows the distribution of GC, AT and N percentages in the same bins as the inner plot. A summary of complete, fragmented, duplicated and missing BUSCO genes in the laurasiatheria_odb10 set is shown in the top right. An interactive version of this figure is available at https://blobtoolkit.genomehubs.org/view/CAUOHF01/dataset/CAUOHF01/snail.

**Figure 3.**
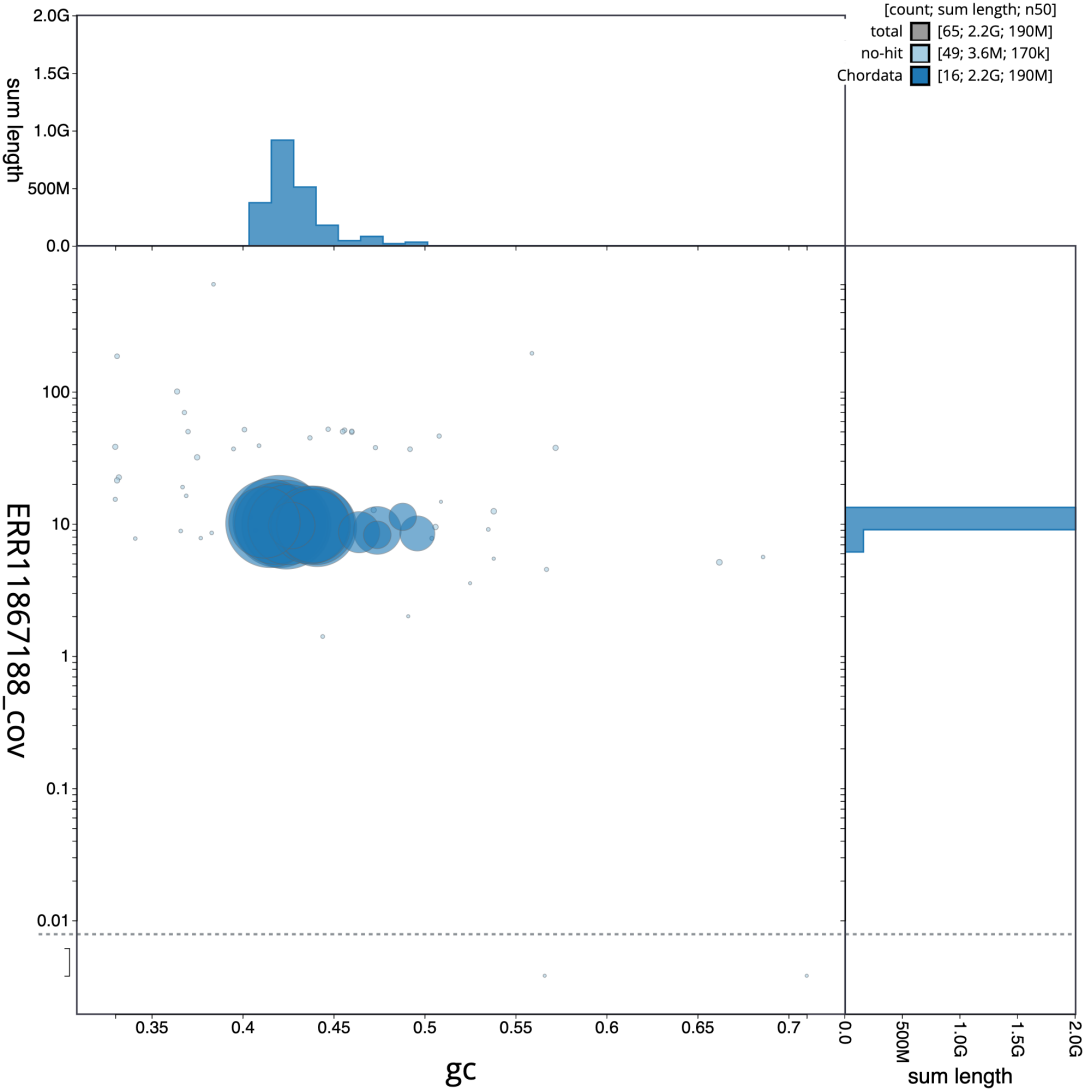
Genome assembly of *Plecotus auritus*, mPleAur1.1: BlobToolKit GC-coverage plot. Sequences are coloured by phylum. Circles are sized in proportion to sequence length. Histograms show the distribution of sequence length sum along each axis. An interactive version of this figure is available at https://blobtoolkit.genomehubs.org/view/CAUOHF01/dataset/CAUOHF01/blob.

**Figure 4.**
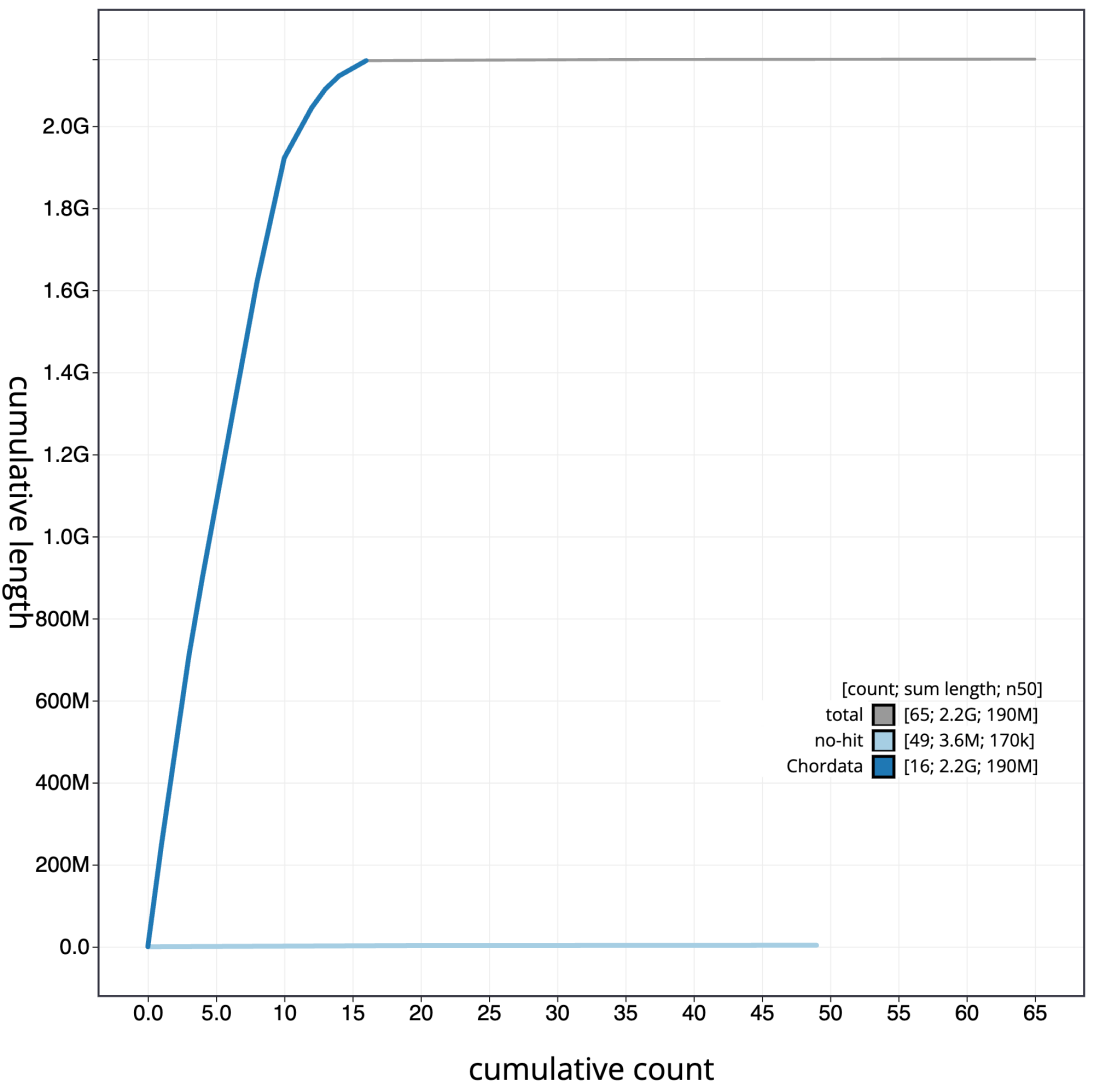
Genome assembly of *Plecotus auritus*, mPleAur1.1: BlobToolKit cumulative sequence plot. The grey line shows cumulative length for all sequences. Coloured lines show cumulative lengths of sequences assigned to each phylum using the buscogenes taxrule. An interactive version of this figure is available at https://blobtoolkit.genomehubs.org/view/CAUOHF01/dataset/CAUOHF01/cumulative.

**Figure 5.**
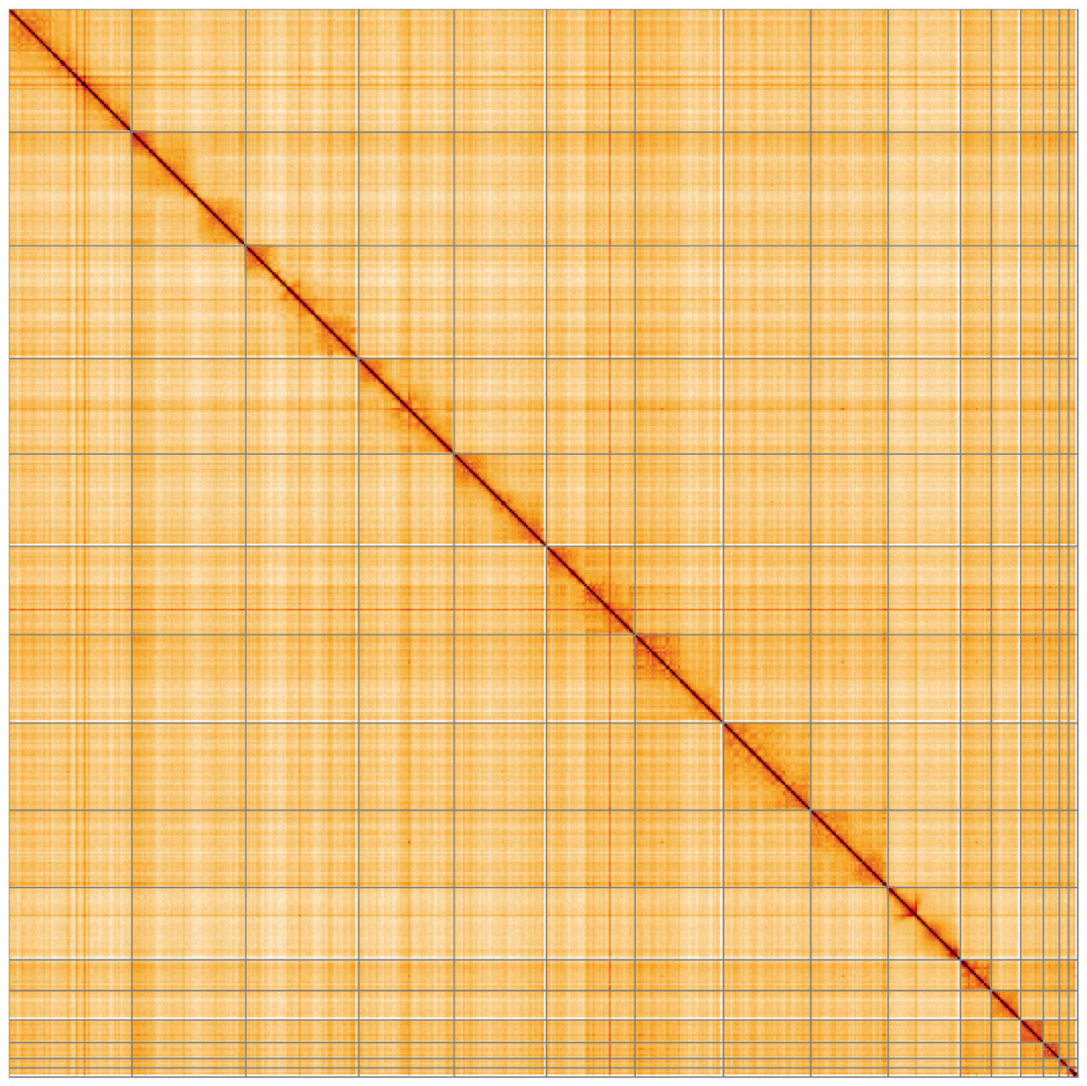
Genome assembly of *Plecotus auritus*, mPleAur1.1: Hi-C contact map of the mPleAur1.1 assembly, visualised using HiGlass. Chromosomes are shown in order of size from left to right and top to bottom. An interactive version of this figure may be viewed at https://genome-note-higlass.tol.sanger.ac.uk/l/?d=BwOAeHE5S6eID4YvhIko0Q.

The final assembly has a total length of 2,163.2 Mb in 64 sequence scaffolds with a scaffold N50 of 186.5 Mb ( Table 1). The snail plot in Figure 2 provides a summary of the assembly statistics, while the distribution of assembly scaffolds on GC proportion and coverage is shown in Figure 3. The cumulative assembly plot in Figure 4 shows curves for subsets of scaffolds assigned to different phyla. Most (99.84%) of the assembly sequence was assigned to 16 chromosomal-level scaffolds, representing 15 autosomes and the X sex chromosome. Chromosome-scale scaffolds confirmed by the Hi-C data are named in order of size ( Figure 5; Table 2). Chromosome X was assigned based on synteny to *Pipistrellus pygmaeus* (GCA_949987585.1) ( Ruedi *et al.*, 2023). While not fully phased, the assembly deposited is of one haplotype. Contigs corresponding to the second haplotype have also been deposited. The mitochondrial genome was also assembled and can be found as a contig within the multifasta file of the genome submission.

**Table 1.** Genome data for *Plecotus auritus*, mPleAur1.1.

Project accession data		
Assembly identifier	mPleAur1.1	
Species	*Plecotus auritus*	
Specimen	mPleAur1	
NCBI taxonomy ID	61862	
BioProject	PRJEB65183	
BioSample ID	SAMEA110056142	
Isolate information	mPleAur1, female (DNA, Hi-C and RNA sequencing)	
Assembly metrics *		*Benchmark*
Consensus quality (QV)	59.8	*≥ 50*
*k*-mer completeness	100.0%	*≥ 95%*
BUSCO **	C:96.1%[S:93.6%,D:2.6%], F:0.7%,M:3.2%,n:12,234	*C ≥ 95%*
Percentage of assembly mapped to chromosomes	99.84%	*≥ 95%*
Sex chromosomes	X	*localised homologous pairs*
Organelles	Mitochondrial genome: 16.91 kb	*complete single alleles*
Raw data accessions		
PacificBiosciences SEQUEL II	ERR11867187, ERR11867189, ERR11867188, ERR11867190	
Hi-C Illumina	ERR11872529	
PolyA RNA-Seq Illumina	ERR11872530	
Genome assembly		
Assembly accession	GCA_963455305.1	
*Accession of alternate haplotype*	GCA_963455325.1	
Span (Mb)	2163.2	
Number of contigs	1302	
Contig N50 length (Mb)	2.9	
Number of scaffolds	64	
Scaffold N50 length (Mb)	186.5	
Longest scaffold (Mb)	248.79	

* Assembly metric benchmarks are adapted from column VGP-2020 of “ Table 1: Proposed standards and metrics for defining genome assembly quality” from Rhie *et al.* (2021). ** BUSCO scores based on the laurasiatheria_odb10 BUSCO set using version 5.3.2. C = complete [S = single copy, D = duplicated], F = fragmented, M = missing, n = number of orthologues in comparison. A full set of BUSCO scores is available at https://blobtoolkit.genomehubs.org/view/CAUOHF01/dataset/CAUOHF01/busco.

**Table 2.** Chromosomal pseudomolecules in the genome assembly of *Plecotus auritus*, mPleAur1.

INSDC accession	Chromosome	Length (Mb)	GC%
OY734022.1	1	248.79	42.0
OY734023.1	2	230.26	42.5
OY734024.1	3	228.11	41.5
OY734025.1	4	192.8	42.0
OY734026.1	5	186.5	42.5
OY734027.1	6	179.15	44.0
OY734028.1	7	178.55	44.0
OY734029.1	8	176.15	43.5
OY734030.1	9	156.2	44.0
OY734032.1	10	62.5	47.5
OY734033.1	11	59.24	42.5
OY734034.1	12	45.6	46.5
OY734035.1	13	32.12	49.5
OY734036.1	14	18.9	47.5
OY734037.1	15	18.61	49.0
OY734031.1	X	146.14	41.0
OY734038.1	MT	0.02	38.5

The estimated Quality Value (QV) of the final assembly is 59.8 with *k*-mer completeness of 100.0%, and the assembly has a BUSCO v5.3.2 completeness of 96.1% (single = 93.6%, duplicated = 2.6%), using the laurasiatheria_odb10 reference set ( *n* = 12,234).

Metadata for specimens, barcode results, spectra estimates, sequencing runs, contaminants and pre-curation assembly statistics are given at https://links.tol.sanger.ac.uk/species/61862.

## Methods

### Sample acquisition and nucleic acid extraction

A female *Plecotus auritus* (specimen ID NHMUK014438751, ToLID mPleAur1) was collected from Ringmer, East Sussex, England, UK (latitude 50.89, longitude 0.05) on 2021-09-25. This specimen was caught by a cat on 4 September 2021 in Ringmer, East Sussex BN8 5HZ and brought to the Hurstpierpoint bat hospital, where its condition was found to be life threatening, with one or more open wounds. Initially feeding, given antibiotics and painkillers, it became weaker, went off its food and developed breathing difficulties. On 25 September it was deemed kindest to euthanise, which was carried out by cervical dislocation. The animal was immediately dissected and its tissue was preserved by freezing. The specimen was collected and identified by Amanda Millar (Sussex Bat Group) and tissues were stored at –80 °C.

The workflow for high molecular weight (HMW) DNA extraction at the Wellcome Sanger Institute (WSI) Tree of Life Core Laboratory includes a sequence of core procedures: sample preparation; sample homogenisation, DNA extraction, fragmentation, and clean-up. In sample preparation, the mPleAur1 sample was weighed and dissected on dry ice ( Jay *et al.*, 2023).

Tissue from the muscles and organs was homogenised using a PowerMasher II tissue disruptor ( Denton *et al.*, 2023a).

HMW DNA was extracted using the Automated MagAttract v1 protocol ( Sheerin *et al.*, 2023). DNA was sheared into an average fragment size of 12–20 kb in a Megaruptor 3 system with speed setting 30 ( Todorovic *et al.*, 2023). Sheared DNA was purified by solid-phase reversible immobilisation ( Strickland *et al.*, 2023): in brief, the method employs a 1.8X ratio of AMPure PB beads to sample to eliminate shorter fragments and concentrate the DNA. The concentration of the sheared and purified DNA was assessed using a Nanodrop spectrophotometer and Qubit Fluorometer and Qubit dsDNA High Sensitivity Assay kit. Fragment size distribution was evaluated by running the sample on the FemtoPulse system.

RNA was extracted from tissue of mPleAur1 in the Tree of Life Laboratory at the WSI using the RNA Extraction: Automated MagMax™ *mir*Vana protocol ( do Amaral *et al.*, 2023). The RNA concentration was assessed using a Nanodrop spectrophotometer and a Qubit Fluorometer using the Qubit RNA Broad-Range Assay kit. Analysis of the integrity of the RNA was done using the Agilent RNA 6000 Pico Kit and Eukaryotic Total RNA assay.

Protocols developed by the WSI Tree of Life laboratory are publicly available on protocols.io ( Denton *et al.*, 2023b).

### Sequencing

Pacific Biosciences HiFi circular consensus DNA sequencing libraries were constructed according to the manufacturers’ instructions. Poly(A) RNA-Seq libraries were constructed using the NEB Ultra II RNA Library Prep kit. DNA and RNA sequencing was performed by the Scientific Operations core at the WSI on Pacific Biosciences SEQUEL II (HiFi) and Illumina NovaSeq 6000 (RNA-Seq) instruments. Hi-C data were also generated from tissue of mPleAur1 using the Arima2 kit and sequenced on the Illumina NovaSeq 6000 instrument.

### Genome assembly and curation

Assembly was carried out with Hifiasm ( Cheng *et al.*, 2021) and haplotypic duplication was identified and removed with purge_dups ( Guan *et al.*, 2020). The assembly was then scaffolded with Hi-C data ( Rao *et al.*, 2014) using YaHS ( Zhou *et al.*, 2023). The assembly was checked for contamination and corrected using the gEVAL system ( Chow *et al.*, 2016) as described previously ( Howe *et al.*, 2021). Manual curation was performed using gEVAL, HiGlass ( Kerpedjiev *et al.*, 2018) and Pretext ( Harry, 2022). The mitochondrial genome was assembled using MitoHiFi ( Uliano-Silva *et al.*, 2023), which runs MitoFinder ( Allio *et al.*, 2020) or MITOS ( Bernt *et al.*, 2013) and uses these annotations to select the final mitochondrial contig and to ensure the general quality of the sequence.

### Evaluation of final assembly

A Hi-C map for the final assembly was produced using bwa-mem2 ( Vasimuddin *et al.*, 2019) in the Cooler file format ( Abdennur & Mirny, 2020). To assess the assembly metrics, the *k*-mer completeness and QV consensus quality values were calculated in Merqury ( Rhie *et al.*, 2020). This work was done using Nextflow ( Di Tommaso *et al.*, 2017) DSL2 pipelines “sanger-tol/readmapping” ( Surana *et al.*, 2023a) and “sanger-tol/genomenote” ( Surana *et al.*, 2023b). The genome was analysed within the BlobToolKit environment ( Challis *et al.*, 2020) and BUSCO scores ( Manni *et al.*, 2021; Simão *et al.*, 2015) were calculated.

Table 3 contains a list of relevant software tool versions and sources.

**Table 3.** Software tools: versions and sources.

Software tool	Version	Source
BlobToolKit	4.2.1	https://github.com/blobtoolkit/blobtoolkit
BUSCO	5.3.2	https://gitlab.com/ezlab/busco
Hifiasm	0.19.5-r587	https://github.com/chhylp123/hifiasm
HiGlass	1.11.6	https://github.com/higlass/higlass
Merqury	MerquryFK	https://github.com/thegenemyers/MERQURY.FK
MitoHiFi	3	https://github.com/marcelauliano/MitoHiFi
PretextView	0.2	https://github.com/wtsi-hpag/PretextView
purge_dups	1.2.5	https://github.com/dfguan/purge_dups
sanger-tol/genomenote	v1.0	https://github.com/sanger-tol/genomenote
sanger-tol/readmapping	1.1.0	https://github.com/sanger-tol/readmapping/tree/1.1.0
YaHS	1.2a.2	https://github.com/c-zhou/yahs

### Wellcome Sanger Institute – Legal and Governance

The materials that have contributed to this genome note have been supplied by a Darwin Tree of Life Partner. The submission of materials by a Darwin Tree of Life Partner is subject to the **‘Darwin Tree of Life Project Sampling Code of Practice’**, which can be found in full on the Darwin Tree of Life website here. By agreeing with and signing up to the Sampling Code of Practice, the Darwin Tree of Life Partner agrees they will meet the legal and ethical requirements and standards set out within this document in respect of all samples acquired for, and supplied to, the Darwin Tree of Life Project.

Further, the Wellcome Sanger Institute employs a process whereby due diligence is carried out proportionate to the nature of the materials themselves, and the circumstances under which they have been/are to be collected and provided for use. The purpose of this is to address and mitigate any potential legal and/or ethical implications of receipt and use of the materials as part of the research project, and to ensure that in doing so we align with best practice wherever possible. The overarching areas of consideration are:

•   Ethical review of provenance and sourcing of the material

•   Legality of collection, transfer and use (national and international)

Each transfer of samples is further undertaken according to a Research Collaboration Agreement or Material Transfer Agreement entered into by the Darwin Tree of Life Partner, Genome Research Limited (operating as the Wellcome Sanger Institute), and in some circumstances other Darwin Tree of Life collaborators.

## Data Availability

European Nucleotide Archive: *Plecotus auritus* (brown big-eared bat). Accession number PRJEB65183; https://identifiers.org/ena.embl/PRJEB65183 ( Wellcome Sanger Institute, 2023). The genome sequence is released openly for reuse. The *Plecotus auritus* genome sequencing initiative is part of the Darwin Tree of Life (DToL) project. All raw sequence data and the assembly have been deposited in INSDC databases. The genome will be annotated using available RNA-Seq data and presented through the Ensembl pipeline at the European Bioinformatics Institute. Raw data and assembly accession identifiers are reported in Table 1.

## Author information

Members of the Natural History Museum Genome Acquisition Lab are listed here: https://doi.org/10.5281/zenodo.7139035.

Members of the Darwin Tree of Life Barcoding collective are listed here: https://doi.org/10.5281/zenodo.4893703.

Members of the Wellcome Sanger Institute Tree of Life Management, Samples and Laboratory team are listed here: https://doi.org/10.5281/zenodo.10066175.

Members of Wellcome Sanger Institute Scientific Operations: Sequencing Operations are listed here: https://doi.org/10.5281/zenodo.10043364.

Members of the Wellcome Sanger Institute Tree of Life Core Informatics team are listed here: https://doi.org/10.5281/zenodo.10066637.

Members of the Tree of Life Core Informatics collective are listed here: https://doi.org/10.5281/zenodo.5013541.

Members of the Darwin Tree of Life Consortium are listed here: https://doi.org/10.5281/zenodo.4783558.
