# Supplementary material for: The genome sequence of the Brown Long-eared bat, Plecotus auritus (Linnaeus 1758)
Source: Wellcome Open Res. 2024 May 9;9:246. [Version 1] doi: 10.12688/wellcomeopenres.21501.1 (PMC11263909; doi:10.12688/wellcomeopenres.21501.1)
Supplement: Peer review history

# Reviewer report: Reviewer response for version 1

**DOI:** 10.21956/wellcomeopenres.23772.r86500

**Published:** 2024-07-22

**Authors:** Michael R Buchalski

**Affiliations:** 1 California Department of Fish and Wildlife, Sacramento, CA, USA

In this manuscript A. Millar and collaborators present a chromosome-length de novo genome assembly for *Plecotus auritus*. In my opinion, the sequencing and assembly methods are comparable to other high-quality assemblies for other non-model organisms, short of telomere-to-telomere assembly. My major comment is that the manuscript would benefit from the addition of basic information on genus/species biology, methodology, and results. I have not reviewed this journal before, so I’m not familiar with your format and philosophy. This manuscript is marked as a ‘Data Note’. From what I found online, there are no stated word/length limits for this type of article. Therefore, I would imagine such information could be added without violating manuscript length limitations **.**

 A good example of my point is the abstract. I could find no limitations on abstracts other than 300 words max. So why is this abstract ~50 words to the exclusion of so much important and relevant information? More specifically, what is the geographic range and listing status of *P. auritus*? What are the characteristics of the genus that make it interesting for genomic investigation? What were the scaffold and contig N50 values for the assembly, or BUSCO scores? Why is this assembly a valuable contribution to bat research?

 The remainder of the manuscript follows this trend of absolute minimalism, where I can find no such requirements in your guidelines to authors for Data Notes. The manuscript would be a more effective read if the authors did not repeatedly refer to internal laboratory protocols published online when methods could be adequately described in a few sentences. And it is insufficient to repeatedly claim that various laboratory kits were used ‘following manufacturer recommendations’. I refer the authors to the several articles describing genome assemblies conducted by the California Conservation Genomics Project published in Journal of Heredity. Dozens of articles implementing similar methods can be found that strike an excellent balance of brevity and detail. As written, it is difficult to fully evaluate what the authors have done.

 Specific comments can be found below:

 Abstract:

- Include information such as geographic range, listing status, interesting characteristics, sequencing technologies, scaffold and contig N50, BUSCO, Why important?

Background:

- Gives simple life history info of the species but nothing about the genus in general. This is a very diverse genus with species distributed throughout Europe, Asia, and Africa. How does a ref genome improve understanding of this genus: evolutionary history as it relates to speciation, local adaptation, etc.?

Genome Sequence Report:

- What are the numbers in parentheses in the first sentence? Coordinates?
- Provide a reference confirming your primary scaffolds conform to the expected number of chromosomes in Plecotus.
- The word synteny is used in different ways these days. You should be more descriptive regarding what you mean and your methods.
- Report all 4 BUSCO scores.
- Organ tissues were collected (albeit homogenized) to sequence the transcriptome and assist with gene annotation. Protocols for QCing the RNA data are described in the methods section. RNA sequencing library prep is described. Where are the results for any of this work presented? How does the reader know if your data is of sufficient quality to be incorporated into the gene annotation process. What is the status of annotation? Who is doing it?

Methods:

- I appreciate that the authors do not take the sacrifice of a bat lightly, but the animal treatment section too long. 
- Last paragraph on Page 4 has grammar issues around ‘Ampure PB beads’.
- Regarding tissue types sampled, be more specific. As written, it sounds as though every muscle and organ type contributed to the sample. I doubt this was the case.   
- It is insufficient to only state that various ‘kits’ were used ‘following manufacturer recommendations’.

Data Availability and Table 1:

- There seems to be some conflation between European Nucleotide Archive and NCBI Genbank nomenclature. The Data Availability section indicates the assembly is archived at the European Nucleotide Archive, yet in Table 1 NCBI descriptors are used (i.e., BioProject, BioSample, NCBI taxonomy ID). The European Nucleotide Archive does not have these designations. Your text and table do not agree. 

Figure 3:

- The authors provide a complete and informative figure caption for Fig 2. This is not the case for Fig 3 onward. The two sentences provided do not help the reader interpret the data. The axes labels (particularly the y-axis) are uninformative.

Figure 4:

- Same comment as above. The authors also introduce a new metric ‘buscogenes taxrule’ for which no description/explanation/reference is given.

Figure 5:

- Same comment as above. The caption would benefit from a brief description of what a contact map depicts, including explanation of horizontal and vertical lines. 

Are sufficient details of methods and materials provided to allow replication by others?

Partly

Is the rationale for creating the dataset(s) clearly described?

No

Are the datasets clearly presented in a useable and accessible format?

Partly

Are the protocols appropriate and is the work technically sound?

Yes

Reviewer Expertise:

Conservation genetics/genomics

I confirm that I have read this submission and believe that I have an appropriate level of expertise to confirm that it is of an acceptable scientific standard, however I have significant reservations, as outlined above.

# Reviewer report: Reviewer response for version 1

**DOI:** 10.21956/wellcomeopenres.23772.r86495

**Published:** 2024-06-21

**Authors:** Xiuguang Mao

**Affiliations:** 1 East China Normal University, Shanghai, China

Millar et al. present a high-quality chromosome-scale assembly for *Plecotus auritus* and the total number of chromosomal-level scaffolds corresponds to the haploid chromosome number of this species (2n=32). This genome provides valuable resource to study the karyotype evolution in Vespertilionidae.

 One minor comment or suggestion:

 In the background section, if the authors can provide more specific and special characteristics about *Plecotus auritus* (e.g. forage, root selection, echolocation and karyotype), it will be better for readers to understand the application of this reference genome.

Are sufficient details of methods and materials provided to allow replication by others?

Yes

Is the rationale for creating the dataset(s) clearly described?

Partly

Are the datasets clearly presented in a useable and accessible format?

Yes

Are the protocols appropriate and is the work technically sound?

Yes

Reviewer Expertise:

evolutionary biology, speciation, comparative genomics, genome assembly, bats

I confirm that I have read this submission and believe that I have an appropriate level of expertise to confirm that it is of an acceptable scientific standard.

# Reviewer report: Reviewer response for version 1

**DOI:** 10.21956/wellcomeopenres.23772.r85484

**Published:** 2024-06-10

**Authors:** Ruiqi Li

**Affiliations:** 1 University of Colorado Boulder, Boulder, Colorado, USA

Miller et al. presented a high-quality genome of the Brown Long-eared bat. While the assembly and annotation of the genome are satisfactory, the Introduction section should include the ecological or evolutionary rationale for sequencing this genome. Additionally, please provide more background and citation on the bat’s less reliant echolocation compared to other species.

Are sufficient details of methods and materials provided to allow replication by others?

Yes

Is the rationale for creating the dataset(s) clearly described?

No

Are the datasets clearly presented in a useable and accessible format?

Yes

Are the protocols appropriate and is the work technically sound?

Yes

Reviewer Expertise:

Genomics

I confirm that I have read this submission and believe that I have an appropriate level of expertise to confirm that it is of an acceptable scientific standard, however I have significant reservations, as outlined above.
